# Gastric glomus tumor: A case report

**DOI:** 10.1186/1477-7819-8-19

**Published:** 2010-03-22

**Authors:** Ioannis Vassiliou, Aliki Tympa, Theodosios Theodosopoulos, Nikolaos Dafnios, Georgios Fragulidis, Andreas Koureas, Evi Kairi

**Affiliations:** 1 Second Department of Surgery, Athens Medical School, Aretaieion Hospital, 76 Vassilisis Sofias Avenue, 11528, Athens, Greece; 2 First Department of Anesthesiology, Athens Medical School, Aretaieion Hospital, 76 Vassilisis Sofias Avenue, 11528, Athens, Greece; 3 First Department of Radiology, Athens Medical School, Aretaieion Hospital, 76 Vassilisis Sofias Avenue, 11528, Athens, Greece; 4 Department of Pathology, Athens Medical School, Aretaieion Hospital, 76 Vassilisis Sofias Avenue, 11528, Athens, Greece

## Abstract

Gastric glomus tumors are rare mesenchymal tumors of the gastrointestinal tract. We describe a 72-year-old patient who presented with episodes of melena and was subsequently investigated for a tumor of the antrum of the stomach. Surgical resection revealed a 2 × 2 × 1.7 cm well circumscribed submucosal tumor, extending into the muscularis propria. The histopathologic examination of the specimen demonstrated a glomus tumor of the stomach. We discuss the preoperative investigation, the diagnostic problems and the surgical treatment of the patient with this rare submucosal lesion.

## Background

Glomus tumors are benign neoplasms of well-differentiated mesenchymal cells. Glomus tumors of the stomach are rare lesions, arising in the intramuscular layer. They typically present as a solitary submucosal nodule in the region of the antrum and pylorus. Preoperative diagnosis of gastric glomus tumors is difficult and requires a multi-faculty medical approach. We present a rare case of a glomus tumor of the stomach along with the investigative procedures and the surgical treatment.

## Case Presentation

Two months ago, a 72-year-old woman presented to her primary care physician with an episode of melena that was suggestive of hemorrhage of the upper gastrointestinal tract. Upon presentation the patient was hemodynamically stable with normal laboratory tests and no evidence of active bleeding in the last 48 hours. Hospitalization was not required and the evaluation was completed in the outpatient department.

**Figure 1:**
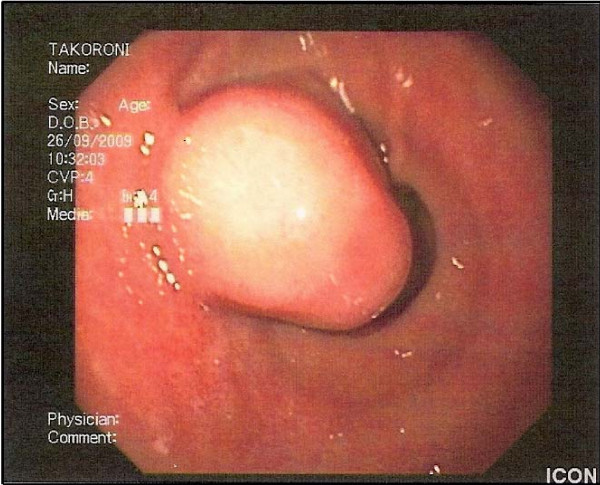
**Glomus tumor of the stomach as featured on upper gastrointestinal endoscopy: a well circumscribed submucosal mass with normal overlying mucosa**.

The patient was subjected to further investigation. Upper gastrointestinal endoscopy revealed mild, diffuse oesophagitis and a small sliding hiatal hernia. At the antrum of the stomach, a 5 cm, well circumscribed submucosal mass with normal overlying mucosa was observed (Figure 1). Multiple regular biopsies were taken and some histological features of leiomyoma were identified. An endoscopic ultrasound confirmed the submucosal lesion which originated from the muscularis propria, measured 1.9 × 2.4 cm and was extending in the second, third and fourth layer of the stomach.

**Figure 2:**
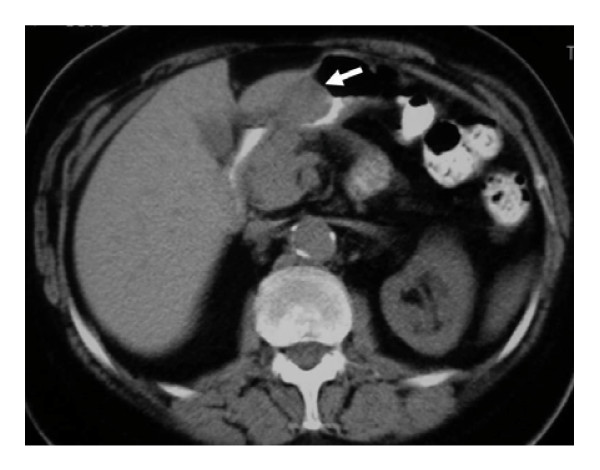
**Glomus tumor of the stomach in a 72 year-old woman: unenhanced computer tomography scan shows the well-circumscribed mass (arrow) in the gastric antrum**.

**Figure 3:**
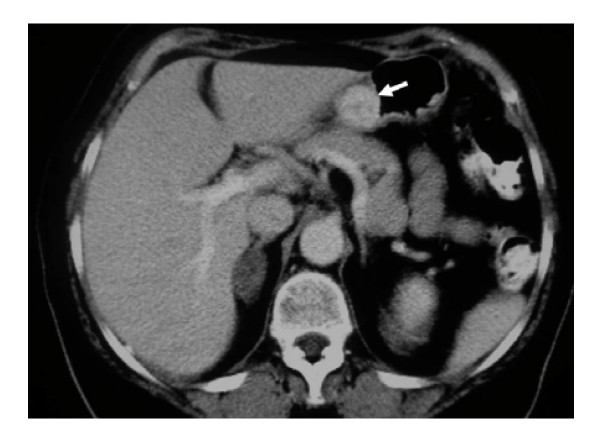
**Glomus tumor of the stomach in a 72 year-old woman: On a contrast-enhanced computer tomography scan, the mass is greatly enhanced (arrow)**.

The patient was subsequently referred for surgical consultation. Physical examination revealed a 72-year-old female who was awake and alert, appeared healthy and looked younger than her stated age. Her abdomen was soft, non-distended, without palpable masses. The stool was negative for occult blood. The hemoglobin level was 13.1 g/dL with normal biochemical profile. Tumor markers were within reference ranges. Abdominal radiography showed normal amount and distribution of gas within the bowel. An abdominal computer tomography scan demonstrated a 3 cm localized, prepyloric enhancing mass at the lesser curvature of the stomach (Figures 2, 3). Lymphadenopathy was not observed. The differential diagnosis involved mesenchymal and other benign gastrointestinal stromal tumors.

**Figure 4:**
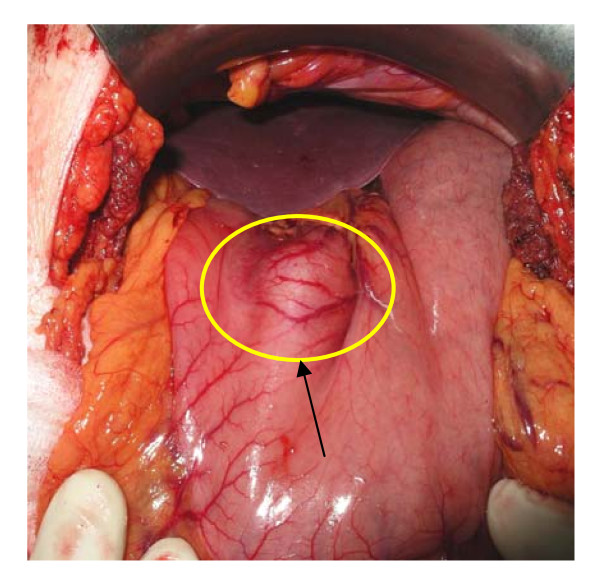
**The prepyloric mass of the stomach at the lesser curvature**.

**Figure 5:**
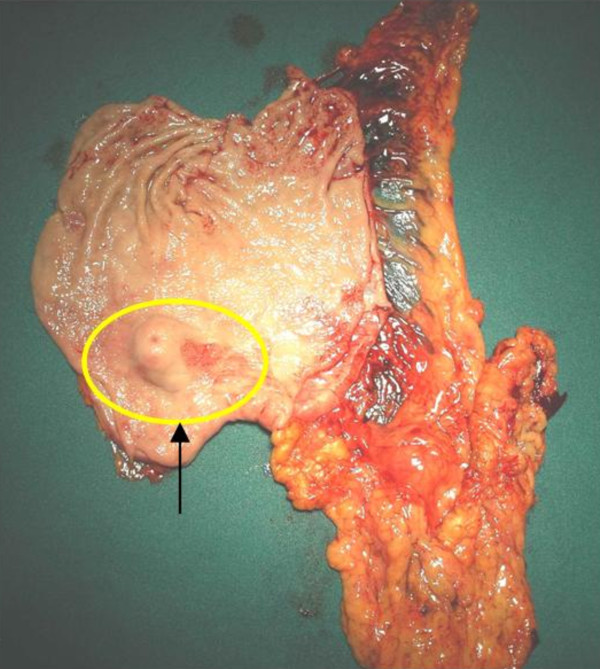
**The specimen of the stomach**.

**Figure 6:**
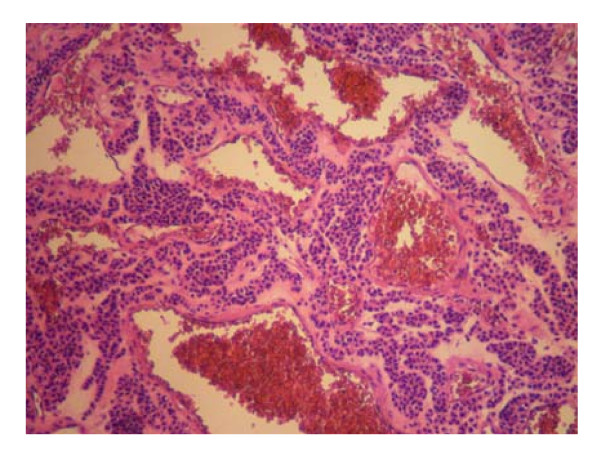
**Trabeculae of tumor cells distributed around dilated and ectactic blood vessels (Hematoxylin & Eosin staining ×100)**.

**Figure 7:**
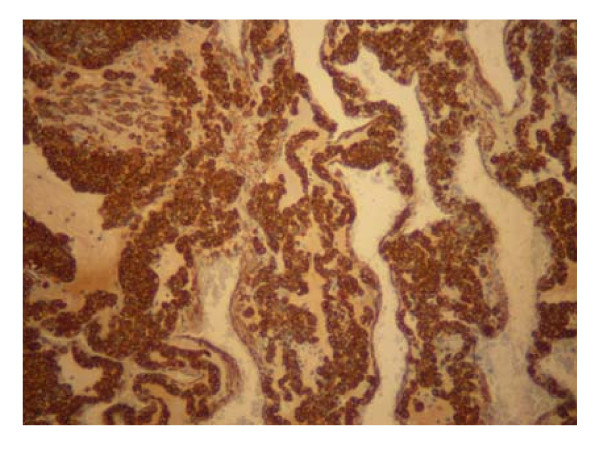
**Glomus tumor of the stomach**. Positive staining for smooth muscle actin (× 100).

The patient was taken to the operative room electively. She was subjected to antrectomy and Roux-en-Y anastomosis. The stomach contained a 2 × 2 × 1.7 cm well circumscribed tumor. (Figures 4, 5). The histopathologic findings of the lesion were characteristic of glomus tumor of the antrum. In detail, cut surface of the specimen, demonstrated a grayish-white nodular tumor, arising from the submucosa and extending through the muscularis of the stomach, without involving the serosal surface. Histologically, the tumor was composed of sheets of glomus cells, without nuclear pleomorphism and no mitotic figures. The cells had eosinophilic and focally clear cytoplasm. Throughout the tumor telengiectatic vessels were observed and some contained aggregates of glomus cells in their walls (Figure 6). Immunohistohemically, the tumor cells were positive for smooth muscle actin (Figure 7) and vimentin and negative for desmin, CD34, CD117, S-100 protein and cytokeratins (AE1/3, CAM 5,2). The proliferating marker Ki-67 was < 5%. The residual gastric mucosa showed atrophic gastritis with focal intestinal metaplasia in the pylorus region. Five lymph nodes retrieved from the major omentum were free of metastatic tumor. The patient recovered uneventfully and was discharged 5 days after surgery.

## Discussion

Gastric glomus tumor is a benign mesenchymal neoplasm arising from the neuromyoarterial glomus. The glomus apparatus consists of three vascular components: an afferent artery separated from an efferent venole by convoluted channels. Multiple layers of epithelioid cells along with nerve fibers surround these channels [1]. Glomus has also been described as an arteriovenous shunt that may contract or expand [2]. Glomus tumors are commonly observed in the dermis or the subcutis. They have also been described in the bone and joints, skeletal muscle, soft tissue, mediastinum, trachea, kidney, uterus and vagina [3].

The first case of gastric glomus tumor was reported in 1951 by Key et al. [4] and since then, few cases have been reported. Vascular tumors of the gastrointestinal tract are rare (accounting for less than 2% of benign tumors), but according to Miettinen et al. [3] the frequency of gastric glomus tumors is estimated to be 1% of that of gastrointestinal stromal tumors. Glomus tumors of the stomach have a marked predominance in females [5-8] although older studies [9] showed nearly equal sex distribution. Moreover, they usually occur in the fifth or sixth decade of life. However, in a clinicopathologic study among Korean population, the age of onset ranged from 30 to 68 years old [7].

Gastric glomus tumors present with a variety of symptoms. Epigastric discomfort (intermittent or continuous), hematemesis, melena and occasionally nausea and vomiting can occur. Overt gastrointestinal bleeding has also been reported [3,7], in cases of ulcerated overlying mucosa. From our literature search, gastric glomus tumors rarely are incidental findings.

Glomus tumors are usually solitary. There is only one case report of multiple gastric glomus tumors [10]. Six glomus tumors were observed in the stomach wall and the perigastric adipose tissue of a 75-year-old black man presenting with hematemesis. Furthermore, gastric glomus tumors are small and have a greater incidence on the greater curvature of the stomach [7,9,11]. In our case, as well as in the report by Yan et al. [12], the tumor occurred in the lesser curvature.

Glomus tumors have to be differentiated from other lesions, such as gastrointestinal stromal tumors (GISTs) and mesenchymal tumors. Preoperative diagnosis of glomus neoplasms is difficult. Glomus tumors grossly appear as red-blue nodules that originate from the muscularis propria [13,14]. In barium studies, most reported cases are localized at the greater curvature side of the antrum and they appear as smooth submucosal masses with or without ulceration. On CT, they manifest as well-circumscribed submucosal masses with homogeneous density on unenhanced study and may contain tiny flecks of calcifications. After contrast medium administration, these tumors show strong enhancement on arterial phase images and persistent enhancement on portal venous phase images, which reflects their hypervascular nature. However, imaging techniques fail to differentiate glomus tumors from other stromal or mesenchymal lesions. The above mentioned imaging features can also be seen with other gastric tumors (endocrine tumors or GISTs). Endoscopic ultrasound findings suggest that gastric glomus tumors are heterogenous, hypoechoic circumscribed masses, with few tubular structures [12,15,16]. They usually originate from the fourth endoscopic ultrasound layer. On Power Doppler sonography, hypervascularity is typical of glomus tumors [3,17]. On the contrary, no turbulent pulsatile flow within leiomyomas was observed [18].

Endoscopic biopsies may fail to provide sufficient amounts of material or representative samples of the submucosal lesion and deeper submucosal lesions cannot be reached adequately [17]. Fine needle aspiration (FNA), performed during endoscopy or endoscopic ultrasound may not contribute to the preoperative diagnosis. In our case, FNA was misleading. Biopsies from the lesion were positive for leiomyoma. Kapur et al. had similar FNA biopsy results [19]. In addition, Lorber et al. [6] reported that FNA biopsy in their case, suggested a well differentiated neuroendocrine tumor, possibly carcinoid. Nevertheless, surgical resection of the tumor and histopathologic examination, demonstrated gastric glomus tumor.

Although glomus tumors of the stomach are usually benign, malignant behavior cannot be excluded. Folpe et al. [13] proposed the following classification criteria for malignant glomus tumors: a) deep location and size more than 2 cm *or *b) presence of atypical mitotic figure *or *c) combination of moderate to high nuclear grade and mitotic activity (5 mitoses/50 high-power fields). It should also be mentioned that the classification criteria have been established for superficial or deep soft tissue glomus tumors. However, due to lack of evidence in the current literature, we suggest that the above mentioned criteria should be used by convention for gastric glomus tumors. Only one case of metastatic gastric glomus tumor has been described [3]. The tumor measured 6.5 cm and on histological analysis mild atypia (1-3 mitoses/HPF) was observed.

Histomorphology of benign gastric glomus tumors is distinctive. Benign glomus tumors consist of small uniform rounded glomus cells that are located in the walls of dilated vessels. The tumor cells have small uniform nuclei, show positive immunoreactivity for smooth muscle actin and are outlined by PAS-positive basement membranes [13]. Glomus tumors are also calponin positive and lack the C-KIT mutation seen with GIST tumors [20]. Immunohistochemistry is essential in the differential diagnosis of glomus tumors. Immunohistochemical staining for actin is negative in gastrointestinal endocrine tumors, but positive in about half of the GISTs. Gastric epithelioid GISTs are usually positive for C-KIT (CD117) [3]. Leiomyomas and leiomyosarcomas are differentiated from GISTs by positive immunoreactivity for desmin and smooth muscle actin and negative immunoreactivity for C-KIT (CD117) and CD34 [8,16].

Finally, operative intervention should be carefully planned in cases of submucosal gastric masses. All the patients with gastric glomus tumors reported in the literature were operated [1-7], [9-16,19]. Lymph node metastases were not common. As gastric glomus tumors are mesenchymal tumors with potential malignant behavior, wedge resection with negative margins should be the treatment of choice [21]. Enucleation is not recommended due to the high recurrence rates [21]. Gastric glomus tumors should always be included in the differential diagnosis of submucosal gastric lesions, keeping in mind that preoperative investigation of these patients often yields misleading results.

## Conclusions

Preoperative diagnosis of gastric glomus tumor is difficult. Despite their distinct histological appearance, their clinicopathologic, radiology and upper endoscopy features overlap with more common gastric tumors. The diagnostic gold standard for such lesions is the histological examination and the immunohistohemical markers. A multi-faculty medical approach of the patient optimizes the chances for an accurate preoperative diagnosis and leads to a targeted surgical intervention.

## Consent

Written informed consent was obtained from the patient for the publication of this case report. A copy of the written consent is available for review by the Editor-in-Chief of this journal

## Competing interests

The authors declare that they have no competing interests.

## Authors' contributions

IV, TT and ND carried out the surgical procedures and contributed to the design of the study. AT gathered the data, drafted the manuscript and critically revised it. AK performed the computed tomography scanning and provided figures for the manuscript along with their interpretation. EK performed the histological analysis of all surgical specimens and provided histological sections as figures of the manuscript. IV revised and finally approved the manuscript for publication.
